# Supplementary material for: Folate Related Pathway Gene Analysis Reveals a Novel Metabolic Variant Associated with Alzheimer’s Disease with a Change in Metabolic Profile
Source: Metabolites. 2022 May 24;12(6):475. doi: 10.3390/metabo12060475 (PMC9230919; doi:10.3390/metabo12060475)
Supplement: Supplementary file 1 [file metabolites-12-00475-s001.zip › metabolites-1734324-supplementary.pdf]

Supplementary Table s1

Heat maps of the effects of SNP variants of known genes found in the neurotransmitter pathway in normal (a) and severe Alzheimer's disease (b) cases. The top row gives the case numbers while the first two columns list the genes and specific SNP site analysed within the gene. Red is homozygous mutant SNP, Yellow is heterozygous which is either neutral or usually have some negative effects, Green is homozygous wild type that is usually positive in function. The clear cells were data points that failed to get SNP data.

a. Normal ageing

Genes	Variants	09_31	11_06	11_25	11_29	12_09	12_11	13_35	14_04	14_08	14_09	14_11	14_20	14_46	15_01	15_28	16_11	16_29	16_31	17_09	17_15	17_34	17_36	18_03	18_11	19_09
**5-HT1A (rs6295)**	1019CG	GG	GG	CC	CC	GC	GC	GC	GC	CC	GG	GC	GC	CC	GG	CC	GC	CC	GC	GC	CC	GC	GG	GC	CC	GC
**5-HT2A (rs6311)**	1438G>A	TT	TT	TC	TC	TT	TC	TC	TC	TC	TC	TC	TT	TC	TC	TT	TC	TC	TC	TC	CC	TC	TC	CC	CC	TT
**ASMT (rs4446909)**		GA	GG	GG	GG	GA	GG	GA	GG	GG	AA	GG	GA	GG	GA	GA	GA	GG	GA	GA	GA	GG	GG	AA	GG	GG
**FKBP5 (rs1360780)**		CT	TT	CC	CT	CT	CT	CT	TT	CC	CC	CC	CT	CC	CT	CC	CT	CC	CT	CT	CT	CT	CT	CC	CT	CT
**IFN-g (rs2430561)**	+874AT	AT	AT	AA	AT	TT	TT	AT	AT	AT	AA	AA	TT	TT	AT	AT	AT	AA	AT	TT	AA	AA	TT	AA	AT	TT
**MAOA (rs6323)**	R297R	TT	TG	TT	TT	TT	TT	TT	TT	TT	TT	TT	TG	TG	GG	GG	TT	TT	TT	TT	TT	TT	GG	TT	TT	TT
**MTNR1B (rs10830963)**		CC	CC	GC	CC	CC	CC	CC	CC	GC	CC	GC	CC	CC	GG	GC	CC	GC	CC	GC	GG	GC	GC	CC	GG	CC
**QDPR (rs1031326)**	690A>G	CC	CC	CT	CT	CT	TT	CC	CT	CC	CT	CT	CC	CC	CT	CT	CT	CT	TT	CC	CC	CT	CT	CT	CC	CC
**SLC18A1 (rs1390938)**	Thr136Ile	GG	GA	GA	GG	AA	GA	GA	GG	GA	GG	GG	GA	GG	GG	GA	GA	GA	GA	GA	GA	GG	GG	GA	GG	GG
**TNF (rs1800629)**	-308GA	GG	AG	AG	GG	GG	GG	AG	GG	AG	AG	GG	AG	GG	GG	AG	GG	GG	AG	AG	AG	GG	AG	GG	GG	GG
**TPH1 (rs1799913)**	A779C	GG	GG	TT	GG	GT	GT	GG	GG	GG	GG	TT	GT	TT	GT	GT	GT	GG	TT	GT	GG	GT	GT	GT	GG	GT
**TPH1 (rs1800532)**	A218C	GG	GG	TT	GG	TG	TG	GG	GG	GG	GG	TT	TG	TT	TG	TG	TG	GG	TT	TG	GG	TG	TG	TG	GG	TG
**TPH2 (rs4570625)**	844G>T	GG	TG	GG	GG	GG	TG	GG	GG	GG	TG	TG	GG	GG	TG	TG	GG	TG	GG	GG	GG	GG	GG	TG	TG	GG
**VDR (rs1544410)**	BsmI	CC	CC	CC	TC	CC	TC	TT	TC	CC	CC	TC	TC	CC	TC	TT	TC	TT	CC	CC	TT	TC	TC	CC	TC	TC
**VDR (rs731236)**	TaqI	AA		AA	GA	AA	GA	GG	GA	AA	AA	GA	GA	AA	GA	GG	GA	GG	AA	AA	GG	GA	GA	AA	GA	GA
**ADRB1 (rs1801253)**	Arg389Gly	CC	CC	CC	CG	CC	CG	CC	CC	CG	CC	CC	CC	CG	CG	CC	CG	CC	CG	CC	CC	CG	CC	CC	CC	CC
**ADRB2 (rs1042713)**	Arg16Gly	GG	AG	AG	AA	AG	GG	AG	AA	GG	AG	AG	GG	AG	AG	GG	GG	AG	GG	AG	GG	AG	AG	AA	AG	AG
**COMT (rs4633)**	H62H	TT	TC	CC	TT	TC	TC	TC	TC	TT	TT	TC	CC	TC	TC	TC	TT	TT		TC	TC	TT	TT	TC	TT	CC
**COMT (rs4680)**	V158M	AA	AG	GG	AA	AG	AG	AG	AG	AA	AA	AG	GG	AG	AG	AG	AA	AA	AG	AG	AG	AA	AA	AG	AA	GG
**DBH (rs1611115)**	C-970T	CC	CT	CC	CC	CC	CT	CC	CC	CC	CC	CC	TT	CC	CC	CC	CC	CT	CC	CC	TT	CC	CT	TT	CT	CC
**DRD2 (rs1076560)**	811-83G>T	CC	CC	CC	CC	CC	CC	AC	CC	CC	AC	AC	CC	CC	AC	AC	CC	AA	CC	CC	AC	CC	AC	CC	CC	CC
**DRD2 (rs6277)**	957C>T	AG	AG	AA	AA	AG	GG	AG	AA	AG	AG	GG	AA	AA	AG	GG	AG	GG	GG	GG	GG	GG	GG	AG	AG	AG
**MAOB (rs1799836)**	A644G	TT	CC	CC	TT	TT	CC	CT	TT	TT	CC		TT	CT	CC	TT	CC	CC	CC	TT	TT		CT	CC	CT	TT
**PNMT (rs876493)**	G-161A	AA	AG	AG	AA	AG	AA	AG	GG	AG	AG	AG	GG	AG	GG	AA	AG	AA	AG	AG	GG	AG	AA	AG	GG	AA
**SLC6A2 (rs5569)**	G1287A	AG	AG	AG	AA	GG	GG	GG	GG	AG	AG	AG	GG	GG	AG	AG	AG	GG	AG	GG	AA	GG	GG	AG	GG	AG
**SLC6A3 (rs27072**	328G>A	CC	CC	CC	CC	CC	CC	CC	TC	CC	CC	CC	CC	TC	CC	CC		CC	CC	TT	TC	CC	TC	TC	CC	TT

**SLC6A3 (rs6347)**		TT	TT	TT	TT	TT	TT	CT	TT	TT	TT	TT	TT	CT	TT	TT	CT	CT	CT	TT	CT	CT	TT	TT	TT	CT
**TH (rs10770141)**	C-824T	GA	GA	GA	GA	GG	GG	GG	AA	GA	GG	GG	GG	GG	GG	GG	GA	GA	GA	GA	GA	AA	GG	GA	GG	GA
**GABRA2 (rs279858)**		CT	CC	CT	CC	TT	CT	CT	CC	CT	TT	TT	CT	CC	CT	CC	CT	TT	CT	CC	CT	CT	CT	CT	TT	CC
**ADRB2 (rs1042713)**	Arg16Gly	GG	AG	AG	AA	AG	GG	AG	AA	GG	AG	AG	GG	AG	AG	GG	GG	AG	GG	AG	GG	AG	AG	AA	AG	AG
**CYP2C19 (rs12248560)**	-806C>T	CC	CC	CC	CC	CC	CC	TC	TC	TC	CC	CC	CC	CC	CC	TT	CC	CC	CC	TC	CC	TC	CC	CC	CC	TC
**CYP2C19 (rs4244285)**	681G>A	GG	GG	AG	AG	GG	GG	GG	GG	GG	GG	GG	GG	GG	GG	GG	GG	GG	AG	GG	GG	GG	GG	GG	AG	GG
**CYP2D6 (rs1135840)**	S486T	GG	GG	GG	CC	CG	GG	CC	GG	CC	CG	GG	GG	CG	GG	GG	CG	CG	CG	CC	CG	CG	CG	CG	GG	CG
**CYP2D6 (rs16947)**	R296C	AA	AA	GA	GG	GG	AA	GG	AA	GG	GA	GA	GA	GA	AA	GG	GA	GA	GA	GG	GA		GA	GA	AA	GG
**CYP2D6 (rs35742686)**	2549DelA	II	II	II	II	II	II	II	II	DI		II	II	II	II	II	II	II	II	II	II	II	II	II	II	II
**CYP2D6 (rs3892097)**	1846G>A	CC	CC	TC	CC	TC	CC	CC	CC	CC	CC	TC	TC	CC	CC	TT	CC	CC	CC	CC	CC	TC	CC	CC	CC	TC
**CYP3A4 (rs2740574)**	-392G>A	TT	TT	CT	TT	TT	TT	TT	TT	TT	TT	TT	TT	TT	TT	TT	TT	TT	TT	TT	TT	TT	TT	TT	TT	TT
**BDNF (rs6265)**	Val66Met	TC	TC	CC	CC	CC	CC	CC	TC	TC	CC	CC	CC	CC	CC	TC	TC	TC	CC	TC	CC	CC	CC	CC	TC	TC
**DIO1 (rs2235544)**	34C>A	AC	CC	CC	AC	AA	AC	AC	CC	AC	AC	AA	AA	CC	CC	CC	AC	AC	AA	AA	CC	CC	CC	AC	AC	AA
**DIO2 (rs12885300)**	Gly3Asp	CC	CC	CC	CC	CC	CC	CC	CC	CC	CC	CC	CC	CC	CC	CC	CC	CC	CC	CC	CC	CC	CC	CC	CC	CC
**DIO2 (rs225014)**	Thr92Ala	TT	CT	CT	TT	CT	TT	CT	TT	TT	CT	TT	CT	CT	CT	CC	TT	CT	TT	CT	CC	TT	CT	TT	TT	TT
**HNMT (i3000469)**	314CT	CC	TT	CC	CC	CC	CC	CC	CC	CC	CC	CC	CC	CC	CC	TC	CC	TC	CC	TC	CC	CC	CC	CC	CC	CC
**OPRM1 (rs1799971)**	A118G	GA	AA	GA	AA	AA	AA	AA	AA	AA	AA		AA	AA	GA	AA	AA	GA	GA	AA	AA		AA	GA	AA	AA
**SLCO1C1 (rs10770704)**	intron3C>T	CT	CT	CT	CT	CT	CC	CC	CT	CC	CT	CT	CT	CT	CT	CC	CT	CT	TT	CC	CC	CC	CC	CT	CT	CT

b. Severe Alzheimer's

Genes	Variants	11_28	12_01	12_03	12_05	12_32	13_09	13_10	13_45	14_10	14_30	14_31	14_50	15_02	15_48	16_10	16_16	16_40	17_37	18_12	18_39	19_04	19_07	19_12	19_29	19_31
**5-HT1A (rs6295)**	1019CG	CC	GC	GG	CC	GC	GC	GC	GC	GC	GC	GC	GC	GC	CC	CC	GC	GG	GC	GC	CC	CC	GG	GC	GC	CC
**5-HT2A (rs6311)**	1438G>A	TT	CC	TT	TC	TC	TC	CC	TC	TT	CC	TC	TC	TC	CC	TC	CC	TC	TC	TT	CC	CC	TC	TC	CC	TC
**ASMT (rs4446909)**		GA	GG	GA	GA	GG	AA	GA	GG	GG	GG	GA	GA	GA	GG	GG	GG	GG	GG	GG	GG	GG	GA	GA	GG	GA
**FKBP5 (rs1360780)**		TT	CT	CT	CT	CC	CC	CT	CC	TT	CC	CC	CC	CC	CT	CT	CT	CC	CT	CC	CC	CT	CC	CT	CT	CC
**IFN-g (rs2430561)**	+874AT	TT	AT	AT	TT	TT	AT	AT	TT	TT	AT	AT	AT	AT	TT	TT	AT	AA	AT	AA	AT	AT	TT	AT	TT	TT
**MAOA (rs6323)**	R297R	TT	TT	GG	TT	GG	TT	TG	GG	TT	TG	GG	TT	GG	TT	TG	TG	GG	TT	TT	TG	TT	TT	TT	TT	TT
**MTNR1B (rs10830963)**		GC	GC	GC	CC	GC	GC	CC	CC	GC	GC	CC	CC	CC	CC	GC	CC	CC	CC	CC	CC	CC	CC	CC	GC	GC
**QDPR (rs1031326)**	690A>G	CT	CC	CT	CC	CT	CC	CC	CC	CT	CT	CT	TT	CC	CT	CC	CC	CC	TT	CT	CC	TT	CT	CC	CT	CC
**SLC18A1 (rs1390938)**	Thr136Ile	GG	GA	GG	GG	GG	GG	GG	GG	GG	GG	GG	GG	GA	GG	GG	GG	GG	GA	GG	GA	GA	GA	AA	GG	GG
**TNF (rs1800629)**	-308GA	GG	GG	GG	GG	AG	AG	AG	GG	AG	GG	GG	AG	GG	AG	AG	GG	GG	GG	AG	GG	AG	AG	AG	GG	GG
**TPH1 (rs1799913)**	A779C	GG	GT	GG	GT	TT	GG	TT	GG	GT	GT	GG	GG	GG	GG	GG	GG	GG	TT	GT	GG	GT	GG	TT	GG	GT
**TPH1 (rs1800532)**	A218C	GG	TG	GG	TG	TT	GG	TT	GG	TG	TG	GG	GG	GG	GG	GG	GG	GG	TT	TG	GG	TG	GG	TT	GG	TG

**TPH2 (rs4570625)**	844G>T	GG	TT	GG	GG	TG	TG	GG	GG	GG	GG	GG	TG	GG	GG	TG	TG	GG	TG	TG	TG	GG	GG	GG	TG	TG
**VDR (rs1544410)**	BsmI	TC	TC	TC	TC	TC	TC	CC	TC	TC	TC	CC	TC	TC	CC	TT	TC	TT	TT	CC	TC	CC	TC	TC	CC	CC
**VDR (rs731236)**	TaqI	GA	GA	GA	GA	GA	GA	AA	GA	GA	GA		GA	GA	AA	GG	GA	GG	GG	AA	GA	AA	GA	GA	AA	AA
**ADRB1 (rs1801253)**	Arg389Gly	CC	CC	CG	CC	CG	CC	CC	CC	GG	CG	CG	CC	CC	CC	CG	CG	CC	CC	CG	CG	CG	CG	CG	GG	CG
**ADRB2 (rs1042713)**	Arg16Gly	GG	GG	AG	AG	AG	AG	GG	AG	GG	AG	AG	AG	GG	AA	GG	GG	AG	AA	GG	AG	AA	GG	GG	GG	AG
**COMT (rs4633)**	H62H	TC	CC	TC	CC	TT	CC	TC	TC	TT	TC	CC	TC	CC	CC	TC	TC	TC	TC	CC	CC	CC	TC	CC	TC	TC
**COMT (rs4680)**	V158M	AG	GG	AG	GG	AA		AG	AG	AA	AG	GG	AG	GG		AG	AG	AG	AG	GG	GG	GG	AG	GG	AG	AG
**DBH (rs1611115)**	C-970T	CT	CC	CC	CC	CT	CC	CT	CT	CC	CC	CC	CC	CC	CC	CT	CC	CC	CT	CT	CC	CT	CC	CT	CC	CC
**DRD2 (rs1076560)**	811-83G>T	CC	CC	CC	CC	CC	CC	AC	AC	CC	CC	AA	AC	CC	CC	AC	CC	CC	AC	CC	CC	CC	AC	CC	CC	AC
**DRD2 (rs6277)**	957C>T	GG	AA	AG	AA	GG	AA	GG	AG	AA	AA	GG	AG	AA	AA	AG	AG	AA	AG	AG	AA	AG	GG	AG	AG	AG
**MAOB (rs1799836)**	A644G	CT	CC	TT	TT	CC	CT	TT	TT	CC	TT	TT	CT	TT	TT	TT	CT	TT	TT	CC	CT	CC	CC	TT	CC	CC
**PNMT (rs876493)**	G-161A	GG	AA	AA	AG	AG	AG	AG	GG	AG	AG	AA	AG	AG	AG	AA	AG	AG	AG	AA	AG	AG	AA	AG	AA	AG
**SLC6A2 (rs5569)**	G1287A	GG	GG	GG	GG	GG	AG	GG	GG	AA	GG	GG	GG	AG	GG	GG	GG	AA	AG	GG	AG	AG	GG	GG	GG	AG
**SLC6A3 (rs27072**	328G>A	CC	CC	CC	CC	CC	TC	CC	CC	CC	TC	TC	CC	CC	CC	CC	CC	CC	CC	CC	CC	CC	CC	TC	CC	CC
**SLC6A3 (rs6347)**		TT	CT	CT	TT	TT	TT	TT	TT	CT	TT	TT	TT	TT	CT	TT	CT	CT	TT	CT	TT	CT	TT	TT	TT	TT
**TH (rs10770141)**	C-824T	GA	GG	GG	GA	GA	GA	GG	GG	AA	GG	GA	GG	GA	GG	AA	GG	GA	GA	GA	GG	AA	GG	GA	GG	GG
**GABRA2 (rs279858)**		CT	CC	CT	CT	CT	CT	CC	CT	TT	CT	CC	TT	CT	CT	CC	CT	TT	TT	TT	CT	CT	CC	CT	CT	CT
**ADRB2 (rs1042713)**	Arg16Gly	GG	GG	AG	AG	AG	AG	GG	AG	GG	AG	AG	AG	GG	AA	GG	GG	AG	AA	GG	AG	AA	GG	GG	GG	AG
**CYP2C19 (rs12248560)**	-806C>T	TC	TC	TC	TC	CC	CC	CC	TT	CC	TC	TC	CC	CC	CC	CC	CC	CC	CC	CC	CC	TC	CC	CC	CC	TC
**CYP2C19 (rs4244285)**	681G>A	AG	GG	GG	GG	GG	GG	AG	GG	GG	GG	GG	AG	GG	AG	AG	GG	GG	AG	GG	AG	GG	GG	GG	GG	AG
**CYP2D6 (rs1135840)**	S486T	CG	GG	CC	CC	CG	CG	CC	CG	CC	CC	CC	CC	CC	GG	CG	CG	GG	CG	GG	CC	CG	CG	GG	CC	GG
**CYP2D6 (rs16947)**	R296C	GG	GG	GG	GG	GA	GG	GG	GG	GG	GG	GG	GG	GG	AA	GA	GG	GA	GA	AA	GG	GG	GA	AA	GG	GA
**CYP2D6 (rs35742686)**	2549DelA	II	II	II	II	II	II	II	DI	DI	II	II	II	II	II	II	II	II	II	II	II	II	II	II	II	II
**CYP2D6 (rs3892097)**	1846G>A	CC	TT	CC	CC	CC	TC	CC	TC	CC	CC		CC	CC	CC	CC	TC	TC	CC	CC	CC	CC	CC	CC	CC	TC
**CYP3A4 (rs2740574)**	-392G>A	TT	TT	CT	TT	TT	TT	TT	TT	TT	TT	TT	CT	TT	TT	TT	TT	TT	TT	TT	TT	TT	TT	TT	TT	TT
**BDNF (rs6265)**	Val66Met	CC	CC	CC	TC	CC	CC	CC	TC	TC	TC	CC	TC	CC	CC	CC	CC	TT	CC	CC	CC	TC	TT	CC	CC	CC
**DIO1 (rs2235544)**	34C>A	AA	AC	AC	AC	AA	AC	AC	AC	AC	AC	AA	CC	AA	AC	AA	AA	AA	AC	AC	AC	AC	CC	CC	AC	AA
**DIO2 (rs12885300)**	Gly3Asp	CC	CC	CC	CC	CC	CC	CC	CC	CC	CC	CC	CC	CC	CC	CC	CC	CC	CC	CC	CC	CC	CC	CC	CC	CC
**DIO2 (rs225014)**	Thr92Ala	TT	CT	CC	CT	CT	TT	CC	TT	TT	CT	CT	TT	CC	CC	TT	CT	CT	CT	CT	CT	CT	TT	CT	CC	CT
**HNMT (i3000469)**	314CT	CC	CC	CC	CC	CC	CC	CC	CC	CC	CC	TC	CC	CC	CC	CC	CC	CC	CC	CC	CC	CC	CC	CC	CC	CC
**OPRM1 (rs1799971)**	A118G	AA	GA	AA	AA	AA	GA	AA	AA	AA	AA	AA	AA	AA	AA	GA	AA	GA	AA	AA	AA	AA	AA	AA	AA	AA
**SLCO1C1 (rs10770704)**	intron3C>T	CC	CT	CC	CC	CT	TT	CT	TT	TT	CT	CT	CT	CT	CT	CT	CC	CT	CC	CT	CT	CC	TT	CT	CC	CC

Supplementary Table s2: The odds of differences of genotypic allelic frequencies of SNPs related to **nervous system** in normal subjects and AD patients: outcome of univariate regression depicted as OR and 95% CI

	Genotype frequencies							Allele frequencies					
**SNPs**	**Genotype**	**Normal**	**AD patients**	**Total**	**P-value**	**OR**	**95% CI**	**Allele**	**Normal**	**AD patients**	**P-value**	**OR**	**95% CI**
5-HT1A (rs6295)	CC	8	7	15		Ref.		C	28	29		Ref.	
	GC	12	15	27		1.43	0.402-5.071	G	22	21	1.000	0.92	0.417-2.035
	GG	5	3	8	0.638	0.69	0.119-3.963						
5-HT2A (rs6311)	CC	3	8	11		Ref.		C	22	29		Ref.	
	TC	16	13	29		0.30	0.067-1.387	T	28	21	0.230	0.57	0.258-1.256
	TT	6	4	10	0.225	0.25	0.040-1.564						
ASMT (rs4446909)	GG	13	14	27		Ref.		G	36	38		Ref.	
	GA	10	10	20		0.93	0.292-2.953	A	14	12	0.820	0.81	0.332-1.989
	AA	2	1	3	0.831	0.46	0.037-5.749						
FKBP5 (rs1360780)	CC	8	12	20		Ref.		C	31	35		Ref.	
	CT	15	11	26		0.49	0.149-1.600	T	19	15	0.527	0.70	0.304-1.607
	TT	2	2	4	0.493	0.67	0.077-5.749						
IFN-g (rs2430561)	TT	7	10	17		Ref.		T	25	33		Ref.	
	AT	11	13	24		0.83	0.236-2.905	A	25	17	0.156	0.52	0.230-1.154
	AA	7	2	9	0.176	0.20	0.032-1.265						
MAOA (rs6323)	TT	19	14	33		Ref.		T	41	33		Ref.	
	TG	3	5	8		2.26	0.462-11.082	G	9	17	0.110	2.35	0.927-5.943
	GG	3	6	9	0.323	2.71	0.577-12.767						
MTNR1B (rs10830963)	CC	14	15	29		Ref.		C	36	40		Ref.	
	GC	8	10	18		1.17	0.358-3.801	G	14	10	0.483	0.64	0.254-1.627

	GG	3	0	3	0.196	1.00						
QDPR* *(rs1031326)	CC	10	12	22		Ref.		C	33	34		Ref.	
	CT	13	10	23		0.64	0.198-2.079	T	17	16	1.000	0.91	0.397-2.104
	TT	2	3	5	0.679	1.25	0.173-9.019						
SLC18A1* *(rs1390938)	GG	11	18	29		Ref.		G	35	42		Ref.	
	GA	13	6	19		**0.28***	0.083-0.959	A	15	8	0.153	0.44	0.169-1.171
	AA	1	1	2	0.118	0.61	0.035-10.79						
TNF* *(rs1800629)	GG	14	14	28		Ref.		G	39	39			
	AG	11	11	22	1.000	1.00	0.327-3.055	A	11	11			
TPH1* *(rs1799913)	GG	10	14	24		Ref.		G	31	35		Ref.	
	GT	11	7	18		0.45	0.131-1.583	T	19	15	0.527	0.70	0.304-1.607
	TT	4	4	8	0.459	0.71	0.143-3.559						
TPH1* *(rs1800532)	GG	10	14	24		Ref.		G	31	35		Ref.	
	TG	11	7	18		0.45	0.131-1.583	T	19	15	0.527	0.70	0.304-1.607
	TT	4	4	8	0.459	0.71	0.143-3.559						
TPH2* *(rs4570625)	GG	16	14	30		Ref.		G	41	38		Ref.	
	TG	9	10	19		1.27	0.402-4.016	T	9	12	0.624	1.44	0.545-3.797
	TT	0	1	1	0.553	1.00							
VDR* *(rs1544410)	CC	10	7	17		Ref.		C	31	29		Ref.	
	TC	11	15	26		1.95	0.564-6.733	T	19	21	0.838	1.18	0.530-2.632
	TT	4	3	7	0.525	1.07	0.180-6.363						
VDR* *(rs731236)	AA	9	6	15		Ref.		A	29	27		Ref.	
	GA	11	15	26		2.05	0.561-7.455	G	19	21	0.836	1.19	0.527-2.675
	GG	4	3	7	0.507	1.13	0.183-6.935						

ADRB1 (rs1801253)	CC	17	11	28		Ref.		C	42	34		Ref.	
	CG	8	12	20		2.32	0.717-7.490	G	8	16	0.100	2.47	0.944-6.464
	GG	0	2	2	0.130								
ADRB2 (rs1042713)	GG	8	11	19		Ref.		G	30	33		Ref.	
	AG	14	11	25		0.57	0.171-1.908	A	20	17	0.679	0.77	0.342-1.744
	AA	3	3	6	0.659	0.73	0.115-4.585						
COMT (rs4633)	TT	9	2	11		Ref.		T	18	33		Ref.	
	TC	12	13	25		4.87	0.872-27.26	C	30	17	**0.008***	3.24	1.415-7.398
	CC	3	10	13	0.016	**14.99***	2.024-111.2						
COMT (rs4680)	AA	9	2	11		Ref.		A	31	17		Ref.	
	AG	13	13	26		4.50	0.810-24.98	G	19	29	**0.024***	2.78	1.217-6.367
	GG	3	8	11	0.036	**12.00***	1.581-91.08						
DBH (rs1611115)	CC	17	16	33		Ref.		C	39	41		Ref.	
	CT	5	9	14		1.91	0.527-6.940	T	11	9	0.803	0.78	0.291-2.082
	TT	3	0	3	0.124	1.00							
DRD2 (rs1076560)	CC	17	17	34		Ref.		C	41	41		Ref.	
	AC	7	7	14		1.00	0.288-3.472	A	9	9	1.000	1	0.360-2.775
	AA	1	1	2	1.000	1.00	0.058-17.33						
DRD2 (rs6277)	GG	9	5	14		Ref.		G	29	21		Ref.	
	AG	11	11	22		1.80	0.455-7.127	A	21	29	0.161	0.52	0.237-1.161
	AA	5	9	14	0.319	3.24	0.690-15.21						
MAOB (rs1799836)	TT	10	12	22		Ref.		T	24	29		Ref.	
	CT	4	5	9		1.04	0.219-4.956	C	22	21	0.682	0.79	0.353-1.770
	CC	9	8	17	0.874	0.74	0.208-2.636						

PNMT* *(rs876493)	AA	7	7	14		Ref.		A	27	30		Ref.	
	AG	13	16	29		1.23	0.343-4.418	G	23	20	0.687	0.78	0.354-1.731
	GG	5	2	7	0.450	0.40	0.057-2.800						
SLC6A2* *(rs5569)	GG	11	17	28		Ref.		G	34	40		Ref.	
	AG	12	6	18		0.32	0.094-1.117	A	16	10	0.254	0.53	0.213-1.324
	AA	2	2	4	0.193	0.65	0.079-5.292						
SLC6A3* *(rs27072)	CC	17	21	38		Ref.		C	39	46		Ref.	
	TC	5	4	9		0.65	0.150-2.794	T	9	4	0.144	0.38	0.108-1.319
	TT	2	0	2	0.285	1.00							
SLC6A3* *(rs6347)	TT	17	17	34		Ref.		T	42	42			
	CT	8	8	16	1.000	1.00	0.305-3.282	C	8	8			
TH* *(rs10770141)	GG	11	12	23		Ref.		G	34	34		Ref.	
	GA	12	10	22		0.76	0.237-2.466	A	16	16	1.000	1	0.360-2.775
	AA	2	3	5	0.808	1.38	0.192-9.834						
GABRA2* *(rs279858)	CC	7	5	12		Ref.		C	27	25		Ref.	
	CT	13	15	28		1.62	0.412-6.338	T	23	25	0.842	1.17	0.535-2.575
	TT	5	5	10	0.788	1.40	0.259-7.582						
ADRB2* *(rs1042713)	GG	8	11	19		Ref.		G	30	33		Ref.	
	AG	14	11	25		0.57	0.171-1.908	A	20	17	0.679	0.77	0.342-1.744
	AA	3	3	6	0.659	0.73	0.115-4.585						
CYP2C19* *(rs12248560)	CC	18	16	34		Ref.		C	42	40		Ref.	
	TC	6	8	14		1.50	0.428-5.259	T	8	10	0.795	1.31	0.471-3.661
	TT	1	1	2	0.817	1.13	0.065-19.49						
CYP2C19* *(rs4244285)	GG	21	17	38		Ref.		G	46	42		Ref.	

	AG	4	8	12	0.185	2.47	0.634-9.625	A	4	8	0.357	0.46	0.128-1.628
CYP2D6 (rs1135840)	GG	10	6	16		Ref.		G	31	21		Ref.	
	CG	11	9	20		1.36	0.356-5.217	C	19	29	0.071	2.25	1.011-5.020
	CC	4	10	14	0.152	4.17	0.894-19.42						
CYP2D6 (rs16947)	AA	6	3	9		Ref.		A	23	12	Ref.	Ref.	
	GA	11	6	17		1.09	0.198-6.007	G	25	38	**0.020***	2.91	1.231-6.895
	GG	7	16	23	0.050	4.57	0.881-23.71						
CYP2D6 (rs35742686)	II	23	23	46		Ref.		I	47	48		Ref.	
	DI	1	2	3	0.576	2.00	0.169-23.623	D	1	2	1.000	0.51	0.045-5.827
CYP2D6 (rs3892097)	CC	18	18	36		Ref.		C	42	41		Ref.	
	TC	6	5	11		0.83	0.215-3.230	T	8	7	1.000	0.90	0.298-2.699
	TT	1	1	2	0.965	1.00	0.058-17.25						
CYP3A4 (rs2740574)	TT	24	23	47		Ref.		T	49	48		Ref.	
	CT	1	2	3	0.552	2.09	0.177-24.615	C	1	2	1.000	0.49	0.043-5.585
BDNF (rs6265)	CC	15	17	32		Ref.		C	40	40			
	TC	10	6	16		0.53	0.155-1.806	T	10	10			
	TT	0	2	2	0.210	1.00							
DIO1 (rs2235544)	AA	6	8	14		Ref.		A	22	30		Ref.	
	AC	10	14	24		1.05	0.277-3.985	C	28	20	0.161	0.52	0.237-1.160
	CC	9	3	12	0.139	0.25	0.047-1.344						
DIO2 (rs12885300)	CC	25	25	50				C	50	50			
DIO2 (rs225014)	TT	12	7	19		Ref.		T	35	27		Ref.	
	CT	11	13	24		2.03	0.592-6.933	C	15	23	0.149	1.99	0.874-4.521
	CC	2	5	7	0.251	4.29	0.650-28.26						

***HNMT* (i3000469)**	CC	21	24	45		Ref.		C	45	49		Ref.	
	TC	3	1	4		0.31	0.030-3.159	T	5	1	0.204	0.18	0.021-1.634
	TT	1	0	1	0.333								
***OPRM1* (rs1799971)**	AA	17	21	38		Ref.		A	40	46		Ref.	
	GA	6	4	10	0.390	0.54	0.131-2.227	G	6	4	0.513	0.58	0.153-2.202
***SLCO1C1* (rs10770704)**	CC	8	8	16		Ref.		C	32	29		Ref.	
	CT	16	13	29		0.81	0.239-2.761	T	18	21	0.682	1.29	0.575-2.881
	TT	1	4	5	0.348	4.00	0.363-44.11						

*statistically significant

Supplementary Table s3: The odds of differences of genotypic allelic frequencies of SNPs related to **methylation** in normal subjects and AD patients: outcome of univariate regression depicted as OR and 95% CI

	**Genotype frequencies**							**Allele frequencies**					
**SNPs**	**Genotype**	**Normal**	**AD**	**Total**	**P-value**	**OR**	**95% CI**	**Allele**	**Normal**	**AD**	**P-value**	**OR**	**95% CI**
ALDH2 (rs671)	GG	25	24	49				G	50	48			
DHFR (rs70991108)	II	12	9	21		Ref.		I	32	31		Ref.	
	ID	8	13	21		2.17	0.631-7.442	D	18	19	1.000	1.09	0.484-2.455
	DD	5	3	8	0.347	0.80	0.150-4.258						
FOLH1 (rs202700)	CC	8	10	18		Ref.		C	32	33		Ref.	
	TC	16	13	29		0.65	0.199-2.122	T	18	15	0.673	0.81	0.349-1.873
	TT	1	1	2	0.774	0.80	0.043-14.89						
MTHFD1 (rs1076991)	CC	8	2	10		Ref.		C	29	13		Ref.	
	CT	13	9	22		2.77	0.473-16.21	T	21	37	**0.002***	**3.93***	1.687-9156
	TT	4	14	18	**0.007***	**14.00***	2.079-94.24						
MTHFD1 (rs2236225)	AA	6	7	13		Ref.		A	26	19		Ref.	
	AG	14	5	19		0.30	0.069-1.364	G	24	29	0.232	1.65	0.742-3.686
	GG	5	12	17	**0.027***	2.06	0.455-9.304						
MTHFR (rs1801131)	TT	12	11	23		Ref.		T	35	31		Ref.	
	GT	11	9	20		0.89	0.268-2.970	G	15	19	0.527	1.43	0.622-3.286
	GG	2	5	7	0.466	2.73	0.436-17.05						
MTHFR (rs1801133)	GG	12	13	25		Ref.		G	34	34			
	AG	10	8	18		0.74	0.219-2.493	A	16	16			
	AA	3	4	7	0.817	1.23	0.227-6.671						
MTR (rs1805087)	AA	17	17	34		Ref.		A	40	41		Ref.	

	GA	6	7	13		1.17	0.324-4.200	G	10	7	0.596	0.68	0.237-1.971
	GG	2	0	2	0.357								
RFC1 (rs1051266)	CC	7	9	16		Ref.		C	27	29		Ref.	
	CT	13	11	24		0.66	0.184-2.350	T	23	21	0.841	0.85	0.386-1.874
	TT	5	5	10	0.812	0.78	0.159-3.795						
SHMT1 (rs1979277)	GG	14	12	26		Ref.		G	37	34		Ref.	
	AG	9	10	19		1.30	0.396-4.242	A	13	16	0.660	1.34	0.563-3.189
	AA	2	3	5	0.816	1.75	0.249-12.28						
TYMS (rs2790)	AA	14	15	29		Ref.		A	36	40		Ref.	
	GA	8	10	18		1.17	0.358-3.801	G	12	10	0.632	0.75	0.289-1.944
	GG	2	0	2	0.327								
AHCY (i5000928)	TT	25	25	50				T	50	50			
BHMT (rs3733890)	GG	13	14	27		Ref.		G	35	38		Ref.	
	AG	9	10	19		1.03	0.319-3.341	A	13	12	0.818	0.85	0.343-2.111
	AA	2	1	3	0.818	0.46	0.037-5.749						
BHMT (rs567754)	CC	11	6	17		Ref.		C	34	27		Ref.	
	TC	12	15	27		2.29	0.656-8.009	T	16	23	0.218	1.81	0.802-4.086
	TT	2	4	6	0.291	3.67	0.513-26.22						
BHMT (rs651852)	TT	6	10	16		Ref.		T	25	31		Ref.	
	TC	13	11	24		0.51	0.139-1.848	C	25	19	0.314	0.61	0.277-1.359
	CC	6	4	10	0.457	0.40	0.079-2.022						
FUT2 (rs1047781)	AA	25	25	50				A	50	50			
FUT2 (rs601338)	AA	4	7	11		Ref.		A	24	28		Ref.	
	AG	16	14	30		0.50	0.121-2.074	G	26	22	0.548	0.73	0.330-1.594

	GG	5	4	9	0.588	0.46	0.076-2.764						
MAT1A (rs1985908)	AA	9	12	21		Ref.		A	30	34		Ref.	
	GA	12	10	22		0.63	0.187-2.085	G	20	14	0.294	0.62	0.266-1.433
	GG	4	2	6	0.533	0.38	0.056-2.519						
MTRR (rs162036)	AA	21	20	41		Ref.		A	45	45			
	GA	3	5	8		1.75	0.369-8.302	G	5	5			
	GG	1	0	1	0.467	-	-						
MTRR (rs1801394)	GG	10	7	17		Ref.		G	32	29		Ref.	
	GA	12	15	27		1.79	0.523-6.100	A	18	21	0.682	1.29	0.575-2.881
	AA	3	3	6	0.650	1.43	0.220-9.262						
PEMT (rs7946)	TT	14	11	25		Ref.		T	36	35		Ref.	
	TC	8	13	21		2.07	0.634-6.750	C	12	15	0.654	1.29	0.528-3.133
	CC	2	1	3	0.394	0.64	0.051-7.965						
TCN2 (rs1801198)	CC	8	5	13		Ref.		C	27	26		Ref.	
	GC	11	16	27		2.33	0.600-9.028	G	21	22	1.000	1.09	0.486-2.433
	GG	5	3	8	0.347	0.96	0.156-5.900						
CBS (rs1801181)	GG	10	13	23		Ref.		G	31	36		Ref.	
	AG	11	10	21		0.70	0.213-2.296	A	19	14	0.395	0.63	0.274-1.471
	AA	4	2	6	0.575	0.38	0.058-2.538						
CBS (rs234706)	GG	13	8	21		Ref.		G	36	29		Ref.	
	AG	10	13	23		2.11	0.632-7.059	A	14	21	0.208	1.86	0.808-4.291
	AA	2	4	6	0.325	3.25	0.480-21.99						
CTH (rs1021737)	GG	18	16	34		Ref.		G	42	40		Ref.	
	TG	6	8	14		1.50	0.428-5.259	T	8	10	0.795	1.31	0.471-3.661

	TT	1	1	2	0.817	1.13	0.065-19.49						
GSS (rs1801310)	GG	11	10	21		Ref.		G	33	33			
	GA	11	13	24		1.30	0.402-4.205	A	17	17			
	AA	3	2	5	0.813	0.73	0.101-5.330						
GSS (rs6088659)	CC	17	17	34		Ref.		C	42	42			
	CT	8	8	16	1.000	1.00	0.305-3.282	T	8	8			
MUT (i6060254)	CC	14	14	28		Ref.		C	36	37		Ref.	
	TC	8	9	17		1.13	0.337-3.760	T	14	13	1.000	0.90	0.373-2.186
	TT	3	2	5	0.879	0.67	0.096-4.623						
SUOX (rs705703)	CC	20	25	45				C	44	50		Ref.	
	TC	4	0	4				T	6	0	**0.027***	**0.07***	0.004-1.238
	TT	1	0	1	0.062								
NOS3 (i6015641)	TT	13	11	24		Ref.		T	36	34		Ref.	
	TC	10	12	22		1.42	0.444-4.531	C	14	16	0.828	1.21	0.514-2.851
	CC	2	2	4	0.840	1.18	0.142-9.827						
NOS3 (rs1799983)	GG	10	9	19		Ref.		G	32	34		Ref.	
	GT	12	16	28		1.48	0.459-4.778	T	18	16	0.833	0.84	0.365-1.916
	TT	3	0	3	0.163								
SOD2 (rs2758331)	AA	10	3	13		Ref.		A	32	19		Ref.	
	AC	12	13	25		3.61	0.798-16.34	C	18	31	**0.016***	**2.90***	1.287-6.535
	CC	3	9	12	**0.033**	**10.00***	1.594-62.73						
SOD2 (rs4880)	GG	10	4	14		Ref.		G	32	20		Ref.	
	GA	12	12	24		2.50	0.611-10.23	A	18	30	**0.027***	**2.67***	1.188-5.986
	AA	3	9	12	0.062	**7.50***	1.307-43.03						

GSTM1 (insert/delete)	DD	15	10	25		Ref.		D	39	34		Ref.	
	II	9	14	23	0.149	2.33	0.733-7.43	I	9	14	0.339	1.78	0.686-4.640
GSTP1 (rs1695)	AA	15	10	25		Ref.		A	38	32		Ref.	
	GA	8	12	20		2.25	0.678-7.47	G	12	18	0.275	1.78	0.747-4.247
	GG	2	3	5	0.368	2.25	0.317-15.97						
GSTT1 (in/del)	II	22	20	42				I	44	40			
APOE	E3/E3	18	6	24		Ref.		E3	42	23		Ref.	
	E3/E4	2	11	13		**16.50***	2.818-96.62	E2	5	1		0.36	0.040-3.319
	E2/E3	4	0	4		1.00		E4	3	26	<0.0001*	**15.83***	4.317-58.02
	E2/E4	1	1	2		3.00	0.162-55.72						
	E4/E4	0	7	7	<0.0001*	1.00							

*statistically significant

Table s4 Mann-Whitney U tests of significance of SNPs associated with normal ageing or Alzheimer's disease. Highlighted genes show significant associations.

a. Variants in the neurotransmitter pathway

	Normal			Alzheimer's												U has a normal distribution with parameters:			Compare to standard normal:	
Genes & variants	R	A	G	R	A	G	n	Rank G	Rank A	Rank R	Normal SUM Ranks	Alzheimer's SUM Ranks	U1	U2	U = min(U1,U2)	Mu	Sigma		z	p
5-HT1A (rs6295)	8	12	5	7	15	3	50	4.5	22	43	630.5	644.5	305.5	319.5	305.5	312.5	46.43544		-0.15075	0.440088
5-HT2A (rs6311)	6	16	3	4	13	8	50	6	26	45.5	707	568	382	243	243	312.5	45.70871		-1.5205	0.064193
ASMT (rs4446909)	13	0	12	14	0	11	50	12	23.5	37	625	650	300	325	300	312.5	44.49977		-0.2809	0.389393
FKBP5 (rs1360780)	2	15	8	2	11	12	50	10.5	33.5	48.5	683.5	591.5	358.5	266.5	266.5	312.5	45.95917		-1.00089	0.15844
IFN-g (rs2430561)	7	11	7	10	13	2	50	5	21.5	42	565.5	709.5	240.5	384.5	240.5	312.5	47.36555		-1.52009	0.064244
MAOA (rs6323)	19	3	3	14	5	6	50	5	13.5	34	701.5	573.5	376.5	248.5	248.5	312.5	43.20838		-1.48119	0.069277
MTNR1B (rs10830963)	3	8	14	0	10	15	50	15	38.5	49	665	610	340	285	285	312.5	44.88079		-0.61273	0.270026
QDPR (rs1031326)	2	13	10	3	10	12	50	11.5	34	48	653	622	328	297	297	312.5	46.57943		-0.33276	0.369656
SLC18A1 (rs1390938)	11	13	1	18	6	1	50	1.5	12	36	553.5	721.5	228.5	396.5	228.5	312.5	44.64143		-1.88166	0.029941
TNF (rs1800629)	0	11	14	0	11	14	50	14.5	39.5	50.5	637.5	637.5	312.5	312.5	312.5	312.5	44.32026		0	0.5
TPH1 (rs1799913)	4	11	10	4	7	14	50	12.5	33.5	46.5	679.5	595.5	354.5	270.5	270.5	312.5	47.20775		-0.88968	0.186818
TPH1 (rs1800532)	4	11	10	4	7	14	50	12.5	33.5	46.5	679.5	595.5	354.5	270.5	270.5	312.5	47.20775		-0.88968	0.186818
TPH2 (rs4570625)	1	10	14	0	9	16	50	15.5	40	50	667	608	342	283	283	312.5	44.01704		-0.67019	0.251367
VDR (rs1544410)	4	11	10	3	15	7	50	9	30.5	47	613.5	661.5	288.5	336.5	288.5	312.5	46.60406		-0.51498	0.303285
VDR (rs731236)	4	11	9	3	15	6	48	8	28.5	45	565.5	610.5	265.5	310.5	265.5	288	43.5885		-0.51619	0.30286
ADRB1 (rs1801253)	17	8	0	11	12	2	50	1.5	12.5	36.5	720.5	554.5	395.5	229.5	229.5	312.5	44.94895		-1.84654	0.032407
ADRB2 (rs1042713)	8	14	3	11	11	3	50	3.5	19	41	604.5	670.5	279.5	345.5	279.5	312.5	46.63416		-0.70764	0.239586
COMT (rs4633)	9	12	3	2	13	10	49	7	26	44	729	496	429	171	171	300	45.75891		-2.81912	0.002408
COMT (rs4680)	9	13	3	2	13	8	48	6	24.5	43	723.5	452.5	398.5	176.5	176.5	287.5	43.8074		-2.53382	0.005641
DBH (rs1611115)	3	5	17	0	9	16	50	17	40.5	49	638.5	636.5	313.5	311.5	311.5	312.5	42.83035		-0.02335	0.490686
DRD2 (rs1076560)	1	7	17	1	7	17	50	17.5	41.5	49.5	637.5	637.5	312.5	312.5	312.5	312.5	41.99125		0	0.5
DRD2 (rs6277)	9	11	5	5	11	9	50	7.5	25.5	43.5	709.5	565.5	384.5	240.5	240.5	312.5	48.10702		-1.49666	0.06724
MAOB (rs1799836)	9	4	10	8	5	12	48	11.5	27	40	583	593	307	268	268	287.5	44.75424		-0.43571	0.331523
PNMT (rs876493)	7	13	5	7	16	2	50	4	22	43.5	610.5	664.5	285.5	339.5	285.5	312.5	45.53256		-0.59298	0.276597
SLC6A2 (rs5569)	11	0	14	17	0	8	50	11.5	22.5	36.5	562.5	712.5	237.5	387.5	237.5	312.5	44.32026		-1.69223	0.045301
SLC6A3 (rs27072)	2	5	17	0	4	21	49	19.5	43	48.5	643.5	581.5	343.5	256.5	256.5	300	36.31635		-1.19781	0.115496
SLC6A3 (rs6347)	0	8	17	1	8	16	50	17	41.5	50	621	654	296	329	296	312.5	42.5		-0.38824	0.348921
TH (rs10770141)	2	12	11	3	10	12	50	12	34.5	48	642	633	317	308	308	312.5	46.57943		-0.09661	0.461518
GABRA2 (rs279858)	7	13	5	5	15	5	50	5.5	24.5	44.5	657.5	617.5	332.5	292.5	292.5	312.5	46.18066		-0.43308	0.332478
CYP2C19 (rs12248560)	1	6	18	1	8	16	50	17.5	41.5	49.5	613.5	661.5	288.5	336.5	288.5	312.5	41.99125		-0.57155	0.283814
CYP2C19 (rs4244285)	0	4	21	0	8	17	50	19.5	44.5	50.5	587.5	687.5	262.5	362.5	262.5	312.5	38.13242		-1.31122	0.094892
CYP2D6 (rs1135840)	4	11	10	10	9	6	50	8.5	26.5	43.5	550.5	724.5	225.5	399.5	225.5	312.5	48.39253		-1.7978	0.036104
CYP2D6 (rs16947)	6	11	7	3	6	16	49	12	32	45	706	519	406	194	194	300	46.07004		-2.30084	0.0107
CYP2D6 (rs35742686)	0	1	23	0	2	23	49	23.5	48	49.5	588.5	636.5	288.5	311.5	288.5	300	20.76656		-0.55377	0.289866
CYP2D6 (rs3892097)	1	6	18	1	5	18	49	18.5	42	48.5	633.5	591.5	308.5	291.5	291.5	300	38.48038		-0.22089	0.412588
CYP3A4 (rs2740574)	0	1	24	0	2	23	50	24	49	50.5	625	650	300	325	300	312.5	21.20418		-0.58951	0.277761
BDNF (rs6265)	0	10	15	2	6	17	50	16.5	40.5	49.5	652.5	622.5	327.5	297.5	297.5	312.5	43.28359		-0.34655	0.364464
DIO1 (rs2235544)	6	10	9	8	14	3	50	6.5	24.5	43.5	564.5	710.5	239.5	385.5	239.5	312.5	47.62738		-1.53273	0.062671
DIO2 (rs12885300)	25	0	0	25	0	0	50	0.5	0.5	25.5	637.5	637.5	312.5	312.5	312.5	312.5	0		#DIV/0!	#DIV/0!
DIO2 (rs225014)	2	11	12	5	13	7	50	10	31.5	47	560.5	714.5	235.5	389.5	235.5	312.5	47.01416		-1.6378	0.050731
HNMT (i3000469)	0	3	22	0	1	24	50	23.5	48.5	50.5	662.5	612.5	337.5	287.5	287.5	312.5	24.22261		-1.03209	0.151014
OPRM1 (rs1799971)	0	6	17	0	4	21	48	19.5	43.5	48.5	592.5	583.5	316.5	258.5	258.5	287.5	34.09155		-0.85065	0.197482
SLCO1C1 (rs10770704)	8	16	1	8	13	4	50	3	20	42.5	663	612	338	287	287	312.5	45.26706		-0.56332	0.286607

b. Variants in the folate and methylation pathways with *APOE* genotyping

	Normal			Alzheimer's												U has a normal distribution with parameters:			Compare to standard normal:	
Genes & variants	R	A	G	R	A	G	n	Rank G	Rank A	Rank R	Normal SUM Ranks	Alzheimer's SUM Ranks	U1	U2	U = min(U1,U2)	Mu	Sigma		z	p
ALDH2 (rs671)	0	0	25	0	0	24	49	25	49.5	49.5	625	600	300	300	300	300	0		#DIV/0!	#DIV/0!
DHFR (rs70991108)	5	8	12	3	13	9	50	11	32	46.5	620.5	654.5	295.5	329.5	295.5	312.5	47.46239		-0.35818	0.360105
FOLH1 (rs202700)	1	16	8	1	13	10	49	9.5	33	48.5	652.5	572.5	327.5	272.5	272.5	300	43.10938		-0.63791	0.261765
MTHFD1 (rs1076991)	4	13	8	14	9	2	50	5.5	21.5	41.5	489.5	785.5	164.5	460.5	164.5	312.5	47.80914		-3.09564	0.000982
MTHFD1 (rs2236225)	6	14	5	7	5	12	49	9	27	43	681	544	356	244	244	300	46.94765		-1.19282	0.11647
MTHFR (rs1801131)	2	11	12	5	9	11	50	12	33.5	47	606.5	668.5	281.5	343.5	281.5	312.5	47.13068		-0.65775	0.255351
MTHFR (rs1801133)	3	10	12	4	8	13	50	13	34.5	47	642	633	317	308	308	312.5	46.83885		-0.09607	0.461731
MTR (rs1805087)	2	6	17	0	7	17	49	17.5	41	48.5	640.5	584.5	315.5	284.5	284.5	300	40.23211		-0.38526	0.350021
RFC1 (rs1051266)	5	13	7	5	11	9	50	8.5	28.5	45.5	657.5	617.5	332.5	292.5	292.5	312.5	47.48791		-0.42116	0.336819
SHMT1 (rs1979277)	2	9	14	3	10	12	50	13.5	36	48	609	666	284	341	284	312.5	46.20827		-0.61677	0.268692
TYMS (rs2790)	2	8	14	0	10	15	49	15	38.5	48.5	615	610	315	285	285	300	43.10938		-0.34795	0.363938
AHCY (i5000928)	0	0	25	0	0	25	50	25.5	50.5	50.5	637.5	637.5	312.5	312.5	312.5	312.5	0		#DIV/0!	#DIV/0!
BHMT (rs3733890)	2	9	13	1	10	14	49	14	37	48	611	614	311	289	289	300	44.00255		-0.24999	0.401299
BHMT (rs567754)	2	12	11	4	15	6	50	9	31	47.5	566	709	241	384	241	312.5	46.15026		-1.54929	0.060656
BHMT (rs651852)	6	13	6	10	11	4	50	5.5	22.5	42.5	580.5	694.5	255.5	369.5	255.5	312.5	47.48791		-1.20031	0.11501
FUT2 (rs1047781)	25	0	0	25	0	0	50	0.5	0.5	25.5	637.5	637.5	312.5	312.5	312.5	312.5	0		#DIV/0!	#DIV/0!
FUT2 (rs601338)	5	16	4	4	14	7	50	6	26.5	46	678	597	353	272	272	312.5	45.16128		-0.89679	0.184917
MAT1A (rs1985908)	4	12	9	2	10	12	49	11	32.5	46.5	675	550	350	250	250	300	45.53256		-1.09812	0.136077
MTRR (rs162036)	1	3	21	0	5	20	50	21	45.5	50	627.5	647.5	302.5	322.5	302.5	312.5	34.36932		-0.29096	0.385542
MTRR (rs1801394)	10	12	3	7	15	3	50	3.5	20	42	670.5	604.5	345.5	279.5	279.5	312.5	46.15026		-0.71506	0.237287
PEMT (rs7946)	14	8	2	11	13	1	49	2	14	37	634	591	334	266	266	300	44.40077		-0.76575	0.221912
TCN2 (rs1801198)	5	11	8	3	16	5	48	7	27	44.5	575.5	600.5	275.5	300.5	275.5	288	43.31969		-0.28855	0.386462
CBS (rs1801181)	4	11	10	2	10	13	50	12	34	47.5	684	591	359	266	266	312.5	46.87423		-0.99202	0.160595
CBS (rs234706)	2	10	13	4	13	8	50	11	33	47.5	568	707	243	382	243	312.5	46.87423		-1.48269	0.069078
CTH (rs1021737)	1	6	18	1	8	16	50	17.5	41.5	49.5	613.5	661.5	288.5	336.5	288.5	312.5	41.99125		-0.57155	0.283814
GSS (rs1801310)	3	11	11	2	13	10	50	11	33.5	48	633.5	641.5	308.5	316.5	308.5	312.5	46.51777		-0.08599	0.465738
GSS (rs6088659)	0	8	17	0	8	17	50	17.5	42.5	50.5	637.5	637.5	312.5	312.5	312.5	312.5	41.64966		0	0.5
MUT (i6060254)	3	8	14	2	9	14	50	14.5	37	48	643	632	318	307	307	312.5	45.64588		-0.12049	0.452046
SUOX (rs705703)	1	4	20	0	0	25	50	23	47.5	50	700	575	375	250	250	312.5	26.80951		-2.33126	0.00987
NOS3 (i6015641)	2	10	13	2	12	11	50	12.5	35.5	48.5	614.5	660.5	289.5	335.5	289.5	312.5	46.21379		-0.49769	0.309352
NOS3 (rs1799983)	3	12	10	0	16	9	50	10	33.5	49	649	626	324	301	301	312.5	45.2135		-0.25435	0.399613
SOD2 (rs2758331)	10	12	3	3	13	9	50	6.5	25	44	759.5	515.5	434.5	190.5	190.5	312.5	47.34669		-2.57674	0.004987
SOD2 (rs4880)	10	12	3	4	12	9	50	6.5	24.5	43.5	748.5	526.5	423.5	201.5	201.5	312.5	47.62738		-2.33059	0.009887
GSTM1 (insert/delete)	15	0	9	10	0	14	48	12	23.5	36	648	528	348	228	228	288	41.97264		-1.4295	0.07643
GSTP1 (rs1695)	2	8	15	3	12	10	50	13	35.5	48	575	700	250	375	250	312.5	46.39422		-1.34715	0.088966
GSTT1 (in/del)	0	0	22	0	0	20	42	21.5	42.5	42.5	473	430	220	220	220	220	0		#DIV/0!	#DIV/0!
APOE	2	19	4	18	7	0	50	2.5	17.5	40.5	423.5	851.5	98.5	526.5	98.5	312.5	45.95917		-4.65631	1.61E-06
